# Climate justice as a public health imperative: a perspective from Sierra Leone considering the 2025 International Court of Justice opinion

**DOI:** 10.1186/s40249-026-01460-7

**Published:** 2026-05-22

**Authors:** Ibrahim Franklyn Kamara, Sia Morenike Tengbe, Morie Vandi, Jacklyne Ashubwe, Binyam Hailu, Bobson Derrick Fofanah, Busi Anissette, Augustus Osborne, Rachel Tolhurst, Timo Minssen

**Affiliations:** 1 Child and Adolescent Health Technical Officer, Family and Reproductive Health, Health Systems and Services Cluster, World Health Organization, 21A-B Riverside Drive, Off Kingharman Road, Freetown, Sierra Leone; 2 Center of Tropical Medicine and Travel Medicine, Department of Infectious Diseases, Amsterdam Infection &Immunity, University of Amsterdam, Amsterdam, The Netherlands; 3 Ministry of Health, 4th and 5th Floors, Youyi Building, Brookfields, Freetown, Sierra Leone; 4 Medwise Solutions, 8th Floor Pinetree Plaza, Kamburu Drive, Off Ngong Road, Nairobi, Kenya; 5 Solthis, 1 The Maze, Off King Street, Wilberforce, Freetown, Sierra Leone; 6 Institute for Development, Western Area, Freetown, Sierra Leone; 7 Liverpool School of Tropical Medicine, Pembroke Place, Liverpool, UK; 8 Centre for Advanced Studies in Bioscience Innovation Law (CeBIL), Faculty of Law, University of Copenhagen, Karen Blixen Plads 16, København S, Copenhagen, Denmark

**Keywords:** Climate change, Extreme weather patterns, Vulnerability, Adaptation, Mitigation, Africa, International Court of Justice, Sierra Leone

## Abstract

Climate change poses an escalating threat to health, livelihoods, and economic development in Sierra Leone, a coastal, low-income country highly dependent on rain-fed agriculture for sustenance and revenue. Rising temperatures, erratic rainfall, floods, landslides, sea-level rise, and coastal erosion disrupt food systems and damage infrastructure, subsequently entrenching poverty, with projected gross domestic product losses of up to 10% by 2050. The health impacts fall disproportionately on children, adolescents and young people, pregnant women, the elderly, and the urban poor, including increased vector- and water-borne diseases, heat-related morbidity, non-communicable diseases, malnutrition, mental illness, and gender-based violence, particularly in informal coastal settlements. Despite these risks, climate adaptation and mitigation remain weakly integrated across sectors. Conflicting policies, fragmented governance systems, inadequate domestic financing, and over-reliance on unpredictable donor support undermine implementation of existing frameworks such as the National Adaptation Programme of Action. An example of policy conflicts is the extractive-sector priorities directly countering those of environmental protection and public health. This correspondence interrogates global and local institutional readiness to mitigate climate-related health risks, drawing on the WHO Operational Framework for Climate-Resilient Health Systems and the multidimensional climate justice approach. Using the July 2025 International Court of Justice advisory opinion on climate change as a legal and moral anchor, we argue for urgent, health-centred, and justice-oriented climate actions. We highlight the need for strengthened cross-sectoral governance, community-level implementation, and legal accountability, particularly in securing international climate financing for adaptation as part of States’ human rights obligations under the United Nations Framework Convention on Climate Change Loss and Damage Framework. We contend that the International Court of Justice opinion provides Sierra Leone with a critical new basis to advocate for international support to protect the fundamental right to health.

## Background

On July 23rd 2025, the International Court of Justice (ICJ) delivered a landmark advisory opinion clarifying the legal obligations of States under international law in the context of climate change. Requested by the United Nations General Assembly following an initiative of the Pacific Island State of Vanuatu, the ICJ opinion emphasized that States have a duty to prevent significant harm to the environment and to protect the human rights of present and future generations [1]. Of particular relevance is the recognition that the right to health is directly threatened by the consequences of climate change, including rising temperatures, extreme weather events, and disease outbreaks [1]. This opinion reinforces the global legal consensus that States must take meaningful action to reduce emissions and support adaptation efforts. For many African countries, such as Sierra Leone, which face acute climate-related health risks despite contributing minimally to global emissions, the ICJ opinion provides a compelling legal and moral basis to advocate for stronger international cooperation and climate justice for health system resilience. Beyond a legal interpretation, this opinion is situated within the multidimensional climate justice discourse, encompassing distributive justice (fair allocation of adaptation resources), procedural justice (inclusive decision-making for vulnerable communities), and recognition justice (acknowledging historical inequities faced by low-emitting nations like Sierra Leone) [2, 3]. This framing aligns with the United Nations Framework Convention on Climate Change (UNFCCC) Loss and Damage discourse, emphasizing the moral and financial responsibility of high-emitting nations to support Sierra Leone’s health-centered climate adaptation measures. Climate justice acknowledges that the people and communities least responsible for climate change, particularly low-income groups and ethnic minorities worldwide, are often the ones who bear its greatest burdens. This context necessitates a more precise examination of the public health impacts of climate change in Africa, using Sierra Leone as a case study.

Climate change refers to the cumulative changes in environmental temperature and weather patterns over time. These changes are a natural phenomenon; however, over the past two centuries, human activities have accelerated the process [4]. Due to the alarming rate of rising ambient temperature, the World Health Organization (WHO) has warned of extreme weather events, including heatwaves, floods, droughts, storms, and an increase in heat-related health conditions [5]. It has been projected that between 2030 and 2050, an estimated 250,000 additional deaths per year are expected from climate-related malnutrition, malaria, diarrhoea, respiratory infections, and heat stress [5].

Climate change inequities are staggering. Approximately 3.6 billion residents of low-income countries, which contribute minimally to global emissions, bear a disproportionate burden of climate change risks [5]. For example, Africa, which emits less than 10% of global greenhouse gases, suffers more severe climate-related impacts on its economy and people as compared to high-emitting continents like Europe and America [6]. Furthermore, climate-related extreme weather events in vulnerable regions like Africa have caused mortality rates more than 15 times those in wealthier regions [5].

Sierra Leone, a coastal low-income West African country with a predominantly rural population, is particularly vulnerable to the impacts of climate change. With a gross domestic product (GDP) per capita of only a few hundred US dollars and heavy dependence on rain-fed agriculture and mining, the country’s economy and livelihoods are highly susceptible to climate variability [7]. The World Bank ranks Sierra Leone among the 15 most climate-affected economies and projects that rising temperatures and erratic rainfall could shrink its GDP by about 10% by 2050. This climate-related impact will worsen pre-existing poverty and inequality, potentially pushing nearly 600,000 more people into poverty by mid-century [8]. Agriculture, which is the backbone of the country’s economy, employs two-thirds of workers and contributes over half of its GDP [7], faces declining productivity as changing rainfall patterns and higher temperatures reduce crop yields and nutritional value [9]. These environmental and economic stresses exacerbate Sierra Leone’s preexisting public health and development challenges, including the legacy of colonial underdevelopment, the brutal civil war (1991–2002), the devastating Ebola epidemic (2014–2016), and a protracted and worsening economic crisis, which have weakened institutions, constrained human capital, and eroded public health systems.

Sierra Leone’s climate vulnerability mirrors that of other low-income coastal nations such as Mozambique, Liberia, and Bangladesh, where rising sea levels, erratic rainfall, and extreme weather events similarly threaten population health and livelihoods [10]. For instance, Mozambique faces comparable challenges from cyclone-induced flooding impacting over 2 million people annually, while Bangladesh contends with saltwater intrusion of arable land affecting agricultural productivity for millions. These comparative cases underscore the shared need for international responsibility and targeted climate financing to protect public health in vulnerable coastal States and reinforce Sierra Leone’s position within a global call for climate justice. This should be done from a point of legal responsibilities, as cited in Article 3 of the UNFCC, which states “developed countries should take the lead in combating climate change and the adverse effects based on equity and in accordance with their common but differentiated responsibilities and respective capabilities” [11].

Against a background of a dearth of evidence on the adverse effects of climate change in Africa, coupled with a lack of mainstreaming of the climate change agenda among low-and-middle-income-countries (LMICs) including Sierra Leone, this correspondence aims to bolster awareness campaigns and advocacy efforts for the government ministries, departments, and agencies, along with the private sector to foreground the climate crisis agenda in Sierra Leone and other sub-Saharan African (SSA) countries, and intentionally invest in creating a climate resilient ecosystem. This can be actualized using the ‘4 A cyclic process’: assess, arrange, access, and action, which forms an integrated global response to climate change [12].

To guide this analysis, we adopt a multidimensional analytical framework that integrates the distributive, procedural, and recognition dimensions of climate justice with the WHO Operational Framework for Climate-Resilient Health Systems [13]. This framework allows us to examine Sierra Leone’s climate change vulnerability not only through a legal lens, as informed by the 2025 ICJ advisory opinion, but also through health system resilience and equitable policy responses. This framework facilitates an exploration of cross-sectoral linkages, such as the interplay between climate science (e.g., temperature and rainfall variability), legal obligations (e.g., ICJ opinion and UNFCCC commitments), and health system responses (e.g., disease surveillance and infrastructure resilience), ensuring a holistic understanding of climate impacts in Sierra Leone. Through this linkage, we aim to address cross-sectoral interactions and position our analysis within the broader discourse of the UNFCCC Loss and Damage framework.

This correspondence documents the correlation between climate change, public health, and climate justice in Sierra Leone, using the 2025 ICJ advisory opinion as a legal and normative anchor. We focus on four questions: (1) how climate impacts worsen health inequities; (2) what governance and policy gaps hinder adaptation and mitigation; (3) how the ICJ opinion can support health-centred climate justice claims; and (4) how knowledge translation can help bridge policy and implementation. To address these questions, we draw on a qualitative synthesis of peer-reviewed literature, grey literature, and relevant national and global policy documents.

## Environmental and economic vulnerability

This section examines environmental and economic vulnerability as a critical dimension of climate change in Sierra Leone, directly linked to public health outcomes and human rights obligations under the ICJ opinion, ensuring relevance to the study’s focus on integrated climate justice. Overall, climate change has already lowered African GDP per capita (1991–2010) by an estimated 13.6% compared to a no-warming scenario [14]. Sierra Leone’s geographical location and topography heighten its climate-related risks. The country lies on the Atlantic coast, with low-lying urban areas and inland mountains. Unregulated deforestation and rapid population growth in informal settlements have eroded natural safeguards such as forests, wetlands, and mangroves. Deforestation on steep hillsides weakens soil stability; subsequently, heavy rains trigger landslides and floods that claim lives, destroy homes, and damage infrastructure. This deforestation is not merely a consequence of large-scale logging; it is also driven by complex local economic pressures, such as the production of charcoal for household energy and income in the absence of affordable, reliable alternatives. These survival strategies, borne from structural poverty, inadvertently weaken the very ecosystems that offer protection from climate shocks, illustrating a vicious cycle that policy must address.

The economy also suffers from the direct costs of climate change-related extreme weather events. Prolonged droughts reduce water supply for hydroelectric power generation; conversely, floods damage roads, bridges, and ports. Rising temperatures impair labour productivity, especially for outdoor workers, further reducing economic output. Economic vulnerability in Sierra Leone is not uniform but varies across socioeconomic strata, gender, and geographic areas. Rural women, for instance, face heightened exposure due to their reliance on rain-fed agriculture and limited access to financial resources for adaptation, while urban poor in informal settlements suffer from infrastructural deficiencies [15, 16]. Coastal regions bear greater risks from sea-level rise compared to inland areas, necessitating disaggregated and targeted policy responses to address differential adaptive capacities. An Intergovernmental Panel on Climate Change (IPCC) assessment projects that increased heat-related loss of labour and flooding from sea level rise will likely worsen in West Africa [14]. The national meteorological datasets from Sierra Leone are limited due to resource constraints; we therefore complement them with global data and qualitative insights from community-level reports and local studies (e.g., Sesay and Osborne [17]) to contextualize vulnerability at sub-national levels, acknowledging the need for future primary data collection to enhance precision.

To mitigate the effects of climate change, Sierra Leone requires a whole-of-government and whole-of-society approach that prioritizes both mitigation and adaptation initiatives. Whereas there is a growing body of evidence on diverse climate mitigation and adaptation strategies, the application of knowledge translation frameworks, such as the knowledge to action (KTA) framework, will promote the adoption of contextually appropriate interventions. Mitigation refers to efforts that reduce or prevent greenhouse gas emissions, such as re-afforestation and protection of forests, promoting renewable energy utilization, and reducing reliance on unsustainable logging. Adaptation focuses on measures that help communities adjust to climate change impacts, such as strengthening flood defenses, diversifying livelihoods, or climate-proofing agriculture. These strategies are in fact mutually reinforcing; for example, reducing deforestation and promoting tree planting not only sequesters carbon dioxide (mitigation) but also improves soil stability, reduces flooding and landslide risks, and enhances community resilience (adaptation). In Sierra Leone, where per capita contributions to global emissions are very small, adaptation must be prioritized alongside mitigation interventions through initiatives like national tree-planting campaigns, household compound greening, and sustainable land-use practices to simultaneously safeguard the environment and strengthen livelihoods. These strategies are conceptualized within the IPCC’s ‘risk-exposure-vulnerability-resilience’ matrix, recognizing feedback loops between land use (e.g., deforestation for charcoal), energy systems (e.g., reliance on fossil fuels), and health outcomes (e.g., air pollution-related diseases), thereby emphasizing the imperative to adopt a systems-based approach to climate action.

## Extreme weather patterns and increased frequency of flooding and landslides

Sierra Leone’s climate has warmed noticeably. Records indicate the country’s average annual temperature has risen by about 0.8 °C over the past five decades [18]. By 2050, average temperatures could be roughly 2.2 °C higher than today [19], far exceeding the 1.5 °C Paris target for temperature increment. While long-term national climate datasets are scarce due to monitoring limitations in Sierra Leone, regional climate analysis from West Africa, supported by IPCC projections, indicates a consistent warming trend of 0.8 °C over five decades and erratic rainfall patterns, providing a provisional empirical basis for our assertions until localized multi-decadal data become available [20]. Heat waves are already more frequent; extreme heat contributes to heat stress, dehydration, and cardiovascular diseases in vulnerable populations [21]. The IPCC reports that hotter, drier conditions will continue to spread across Africa, making many regions “unprecedentedly hot” unless greenhouse gas emissions are cut [22]. Excess heat also intensifies energy demand and strains power supplies during peak periods, which can disrupt healthcare and industry.

Furthermore, rainfall patterns have become increasingly erratic. Sierra Leone’s wet season has seen shorter bursts of heavy rain followed by longer dry spells. Climate models project increasingly unpredictable rains in West Africa [22]. This translates to intense downpours that cause flash floods on one hand, and droughts that threaten crops and water access on the other. For instance, a long dry period early in 2019 resulted in water scarcity for farming communities; this was followed by unusually heavy rains in early August, which caused flash flooding across Freetown, resulting in at least six deaths and the displacement of hundreds of households [23]. This scenario illustrates how these two weather extremes compound each other’s effects, especially in rural areas where farming is the predominant livelihood source. The country needs to employ measures that reduce the pace of temperature increase to prevent further disastrous events, including high morbidity and mortality rates. These measures include a shift from using fossil fuels to hydroelectric and solar-generated plants for electricity generation. Furthermore, investments to improve the public commuting transport system, like buses, can reduce the number of private vehicles that ply the road daily, subsequently reducing total carbon dioxide (CO_2_) emissions. These proposals align with Sierra Leone’s Nationally Determined Contributions (NDCs) under the Paris Agreement, which prioritizes renewable energy targets (e.g., 30% renewable energy by 2030) and the National.

Adaptation Plan of Action (NAPA), which emphasizes disaster risk reduction through infrastructure upgrades; however, gaps persist in funding and local implementation capacity [24].

In addition to structural destruction, heavy floods contaminate water supplies and destroy crops, undermining food security and health. Floodwater creates breeding grounds for mosquitoes, increasing malaria transmission risk. They also trigger the spread of cholera and other diarrheal diseases by polluting drinking water. Over time, recurring floods and landslides damage roads, schools, and farms, eroding economic development and deepening poverty. While attribution science linking specific events to anthropogenic climate change is nascent in Sierra Leone, global studies suggest that non-climatic drivers such as deforestation (driven by charcoal production), unplanned urban expansion, and governance failures in land-use planning amplify the impact of climate-induced floods and landslides, necessitating integrated risk management [25]. Addressing these risks requires a coherent urban development strategy that balances the rights of rural–urban migrants to secure housing and livelihoods with the need to protect the environment. This means investing in proper drainage systems, including stormwater drains, and supporting re-greening strategies such as tree planting to stabilize hillsides and reduce landslide risks. Strengthening the national early warning system is also critical. This would require close multisectoral collaboration between meteorological services, urban planners, health and environmental agencies, communities, and research institutions, to ensure that weather data is sufficiently granular and locally relevant, emergency preparedness plans are in place, and communication channels enable timely response. Without such coordinated investments, extreme rainfall events whose impacts differ across informal and formal settlements will continue to outpace Sierra Leone’s capacity to respond, leaving the most vulnerable populations at greatest risk.

## Worsening inequity in informal settlements

Sierra Leone is undergoing rapid urbanization, particularly in Freetown and regional headquarters such as Bo, Kenema, Makeni, and Port Loko [26]. However, much of this urban growth, as in other SSA countries, has occurred in an unplanned and unregulated manner, leading to the proliferation of informal settlements. These areas are typically characterized by high population density, substandard housing, poor access to basic amenities such as water, sanitation, electricity, and healthcare services, and a lack of secure land tenure [27]. The most notable informal settlements in Sierra Leone include Kroo Bay, Susan’s Bay, and Moa Wharf in Freetown, all of which are located in low-lying coastal areas. These locations make them especially vulnerable to climate-related hazards, including sea-level rise, coastal erosion, intensified rainfall, and frequent flooding [28]. Although comprehensive geospatial modeling is constrained by data availability in Sierra Leone, preliminary vulnerability mapping by local NGOs indicates that over 60% of Freetown’s informal settlements, including Crab Town, Kolleh Town, and Grey bush are in high-risk flood zones, underscoring the urgent need for spatial analysis to inform adaptive urban design and prioritize interventions in the most exposed areas.

Sea-level rise poses an escalating threat to coastal Sierra Leone. While the country lacks long-term tide gauge data, global projections estimate that sea levels could rise by between 1.1 and 1.7 m by the year 2100 under high-emission scenarios, potentially endangering up to 2 million people in coastal and peri-urban areas [29]. Consequences include permanent inundation of low-lying settlements and farmlands, saltwater intrusion of soil and freshwater sources, and loss of critical fishery habitats. Coastal erosion is already affecting communities, with implications for forced relocation, declining agricultural productivity, and collapsing fisheries that threaten both income and food security [6].

Climate change further exacerbates existing vulnerabilities in informal settlements. The poor drainage systems result in flash floods even during moderate rainfall, while stagnant water encourages outbreaks of waterborne diseases such as cholera [30]. These densely populated areas often lack green spaces and ventilation, and function as urban heat islands, intensifying the impact of heat waves [31, 32]. Inadequate waste management leads to pollution of waterways and increases the burden of climate-sensitive diseases [33]. Social inequalities within these communities, associated with gender, age, education, and occupation, compound these risks. By adopting a Gender-Responsive Climate Action framework [34], we recognize that intersecting vulnerabilities such as gender, disability, and age shape adaptive capacity in informal settlements. Women and girls, often tasked with water collection and caregiving, face disproportionate risks during floods, while elderly and disabled individuals struggle with mobility during evacuations. Residents of informal settlements are often excluded from urban planning processes and lack access to formal emergency response systems [35, 36]. Their limited adaptive capacity stemming from poverty, insecure land tenure, and political marginalization means that even small climate shocks can result in devastating long-term consequences [37]. These scenarios highlight the need to adopt targeted, inclusive adaptation strategies under the GEDSI (Gender Equality, Disability, and Social Inclusion) policy.

As sea levels continue to rise, these marginalized communities face disproportionate exposure to displacement, food insecurity, and worsening poverty. The profound inequities faced by residents of informal settlements are not merely a domestic policy challenge; they represent a critical failure in climate justice. Their heightened risks, directly linked to historical global emissions, underscore the human rights dimension of climate change affirmed by the ICJ opinion. Addressing these challenges requires inclusive climate action that combines community participation, settlement upgrading, targeted protection of vulnerable groups, and, where necessary, voluntary and dignified relocation from high-risk zones. These priorities align with Sustainable Development Goal (SDG) 11 and the New Urban Agenda [38], but they also reflect a broader climate justice concern: the populations facing the greatest climate-related risks are often those least responsible for global emissions. In this context, the ICJ opinion strengthens the argument that international climate finance should support settlement adaptation and protection of vulnerable communities as part of a rights-based response to foreseeable harm.

## Reduced crop production

Climate change is undermining agriculture in Sierra Leone. Over 65% of Sierra Leoneans work in subsistence farming [7]. Climate shocks, including drought, flooding, and heat, along with pests, sharply reduce crop yields. IPCC analyses show that agricultural productivity growth in Africa has declined by ~34% due to climate change effects, more than in any other region [6]. Crops like rice and cassava yield less under erratic rains and higher temperatures, and rising carbon dioxide (CO_2_) levels can paradoxically reduce the protein content of these staples [39]. In rain-dependent farming areas of Sierra Leone, unpredictable precipitation has already led to repeated crop failures, whereas saltwater intrusion threatens staple crops. 2022 data from Sierra Leone showed that 81% of households experienced food insecurity, and 26% of children were chronically stunted [40], figures that already reflect the combined pressures of poverty, weak food systems, and a changing climate. These figures are derived from UNICEF, providing a robust temporal snapshot of food insecurity and stunting prevalence, though longitudinal data are needed for trend analysis. Without decisive adaptation measures, these conditions are projected to worsen, as rising temperatures and increasingly erratic rainfall further reduce yields, degrade nutrition, and deepen hunger. Beyond production, climate change disrupts Sierra Leone’s food systems by affecting value chains, market access, and social protection mechanisms. Erratic rainfall delays harvests, disrupting supply to local markets, while floods damage transport infrastructure, increasing food prices and limiting access for rural communities [41]. Integrating food system resilience through diversified livelihoods and safety-nets into adaptation planning is critical to mitigate these cascading impacts on nutrition and health. The result is a vicious cycle where climate shocks compound structural vulnerabilities, pushing more households into crisis and further straining fragile health and social systems. The USAID commercial guide notes that 75% of Sierra Leone’s arable land is uncultivated, a lost opportunity made worse if climate change exacerbates poor yields [42].

Declining agricultural productivity threatens both GDP and food security. Reduced crop output diminishes exports of palm oil and cacao and raises food import bills [43]. Promoting climate-smart agriculture, such as drought-resistant crop varieties, small-scale irrigation technologies, and indigenous knowledge systems for soil management, offers viable adaptation pathways, as evidenced by successful practices in other African contexts [44, 45], and should be prioritized in Sierra Leone’s agricultural policy. Domestically, higher food scarcity pushes up commodity prices and increases malnutrition. According to the 2024 Global Hunger Index (GHI), Sierra Leone ranks 117th out of the 127 countries for which sufficient data were available to compute GHI scores. The country’s GHI score of 31.2 indicates a serious level of hunger, reflecting persistent food insecurity and undernutrition challenges [46]. Chronic malnutrition is detrimental to the immune system and child health and development, adding to the country’s health burden [47]. Climate-change-driven crop losses also reduce household incomes, trapping families in vicious cycles of poverty [48]. Sierra Leone should prioritize climate-smart agriculture, including drought-resistant crops, small-scale irrigation, soil-management practices, and nutrition-sensitive community interventions such as kitchen gardens. These measures would strengthen food security, reduce malnutrition, and protect child development. Because climate-related food insecurity threatens the rights to health and life, the ICJ opinion also strengthens the case for international financing of climate-resilient agriculture and food systems in vulnerable countries.

## Increase in certain health conditions across the life course

The health impacts of climate change in Sierra Leone are analyzed through the WHO’s Building Blocks of Health Systems framework (service delivery, health workforce, financing, information, medicines, and governance) to ensure structural coherence and global comparability [49]. Climate change effects amplify health challenges in Sierra Leone, whose health system, like many other SSA countries, is not well equipped to absorb climate shocks [50]. Furthermore, this exerts further strain on a system situated in a country prone to infectious disease outbreaks, which disrupt routine provision of essential health services. More alarming is the fact that women and children are at heightened risk during outbreaks. Similarly, with climate change, children, pregnant women, the elderly, the disabled, and the poor suffer most from climate-related health effects [5]. It has been postulated that vector-borne and waterborne diseases will increase, exacerbating malnutrition and non-communicable diseases, and worsening maternal and child health outcomes. Some specific health effects are detailed below. These health effects are not linear but interdependent, mediated by intermediary determinants such as air quality (e.g., dust from droughts exacerbating respiratory conditions), food systems (e.g., crop failures driving malnutrition), and migration (e.g., displacement increasing disease exposure). Modeling causal pathways, as suggested by global studies [51, 52], reveals complex interactions that require integrated health-climate responses rather than siloed interventions. Mitigating adverse outcomes in such polycrises scenarios calls for leveraging knowledge management and translation approaches that foster the timely exchange of robust, accessible, and actionable information between relevant stakeholders.

### Increase in non-communicable diseases (NCDs)

Evidence has suggested that rising temperatures cause heat stress, which can trigger cardio-respiratory problems, including heart failure and asthma, and contribute to the growing burden of non-communicable disease (NCDs) in Sierra Leone [53]. Other environmental factors, like the burning of charcoal for domestic purposes and carbon monoxide emissions from old vehicles, further exacerbate NCD-related problems. The resultant indoor air pollution from biomass cooking, combined with drought-related dust, also exacerbates chronic lung diseases [54]. Studies have documented a high prevalence rate of hypertension, diabetes, and mental disorders among the working population, and climate change is expected to intensify this burden. While health education and promotion can help individuals adopt healthier behaviours, such as reducing tobacco use, improving diets, and increasing physical activity, structural approaches are equally necessary, particularly in reducing air pollution through stricter regulation and cleaner energy alternatives [55]. Investment in environmentally friendly initiatives, including improved public transport, safer roads, and green spaces, can encourage physical activity and facilitate lifestyle modifications, thereby helping to mitigate the projected rise in NCDs while also protecting the environment.

### Vector- and water-borne diseases

Warmer temperatures and flooding alter disease distribution. Anopheles mosquitoes, the vector for malaria parasites, the leading cause of under-five death in SSA, thrive in warm and wet conditions. As more areas progressively become warmer, they favour breeding of the vector, facilitating the spread of the disease to new regions [56]. Consequently, an increase in the incidence of malaria in Sierra Leone would likely heighten malaria-related under-five mortality, reversing gains already made and undermining planned interventions to reduce child deaths. There is therefore an urgent need for the Ministry of Health and the Ministry of Environment and Climate Change to co-create adaptation measures to limit vector proliferation. Such measures could include improved drainage systems to reduce stagnant water, increased indoor residual spraying, distribution of insecticide-treated bed nets, and strengthened vector and disease surveillance.

### Increased child mortality, malnutrition, and stunting

In Sierra Leone, malnutrition is one of the main underlying factors for under-five mortality. Furthermore, stunting has been a great challenge, with a prevalence of 5%, which is higher than in other countries in Africa [40]. Additionally, children may experience a greater risk of mental disorders, other malnutrition conditions, infectious diseases, allergy conditions, and respiratory diseases as they are more susceptible to climate-sensitive conditions. This will likely result in a drastic increase in child morbidity and mortality rates. Although local data are scarce, studies have shown that an increase of 1 °C in the minimum daily temperature above 23.9 °C was associated with up to a 22.4% rise in the risk of infant mortality [57]. As already highlighted, climate-change-driven crop failures and food price shocks worsen malnutrition, especially in children [47], further deterring their optimal physical and cognitive development, which deprives them of the opportunity to contribute meaningfully to the country’s economy in the long term. This creates the imperative for the Sierra Leone government, facilitated by developed countries, to invest in early childhood development initiatives and other climate change adaptation and mitigation strategies, not as aid but as a moral obligation for high-emission nations. There is a need for the country to embark on food production and, at the same time, expand the health system and equip health care workers with appropriate knowledge, skills, and competencies to monitor and treat maternal and child malnutrition.

### Derangement in adolescent health and well-being

Despite the critical importance of this stage of life, the health and well-being of adolescents and young people have been neglected for a very long time. The world is rapidly changing, and so are the needs of adolescents and young people. Like all other cohorts, the health and well-being of adolescents will be affected by climate change [58]. The second *Lancet Commission on Adolescents’ Health and Well-being* highlights the adverse effects of climate change and stresses the urgency of implementing interventions sooner rather than later to help adolescents adapt to and cope with its effects [59]. Evidence has shown that climate change will lead to an increase in depression and anxiety among adolescents and young people due to the socioeconomic consequences [58], and exacerbate respiratory infections, malnutrition, gender-based violence, and intimate partner violence, which is already high among adolescents [60]. Beyond depression and anxiety, climate change contributes to eco-anxiety and post-disaster trauma among adolescents, driven by displacement, loss of livelihoods, and uncertainty about the future. Global literature highlights a rising burden of mental health challenges in climate-affected regions, necessitating psychosocial support services and community-based interventions to address these under-recognized impacts in Sierra Leone [61]. In the development of climate change adaptation and mitigation policies and guidelines, adolescent and young people’s health should be mainstreamed. Investing in adolescent health and wellbeing is the right thing to do as it will benefit them now, in their adulthood, and their future children. The negative mental and physical health impacts on adolescents constitute a violation of their right to health and a secure future. The ICJ opinion’s focus on intergenerational equity is particularly relevant here, providing a legal imperative for climate policies that not only mitigate harm but also actively invest in the well-being and adaptive capacity of future generations. Involving adolescents and young people in policymaking and the development of climate change mitigation and adaptive strategies is no longer just good practice, but a step towards fulfilling these fundamental obligations.

### Poor pregnancy outcomes

Women are highly vulnerable to global public health threats. Climate change will worsen maternal health due to food insecurity that may result from drought or excess rainfall. Undernutrition and other climate-related constraints will have a huge impact on pregnancy outcomes. Heat stress in pregnancy can lead to low birth weight and preterm births. Floods and droughts increase women’s food insecurity, which compromises prenatal nutrition [62, 63]. Undernutrition contributes to anaemia, which hinders fetal growth and development. In the context of Sierra Leone, which has a high maternal mortality rate, as a climate-adaptive measure, there is a need for heightened vigilance among healthcare workers to prevent maternal malnutrition and anaemia and initiate appropriate therapy in diagnosed cases. This should be emphasized by the national antenatal care (ANC) guidelines.

## Implications for antimicrobial resistance (AMR) and infection prevention and control (IPC)

Climate change accelerates antimicrobial resistance (AMR) by altering microbial selection, dispersal, and human–animal–environment contact, stressing infection prevention and control (IPC) systems. Climate-driven changes create direct selection and transmission pathways that favour resistant microbes and mobilize resistance genes, according to systematic review evidence linking AMR and climate hazards [64]. Rising ambient temperatures, extreme rainfall and floods, and altered human–animal–ecosystem interactions are repeatedly identified as drivers that increase bacterial growth rates, mobilize contaminants, and expand opportunities for horizontal gene transfer; consequently, this increases the need for robust IPC interventions in healthcare [65, 66]. While IPC activities seek to ensure the safety of processes in health care, the attendant tasks, such as incineration and sterilization, can substantially increase healthcare waste and pollution, contributing to a high carbon footprint [67, 68].

Climate impacts threaten IPC capacity, facility resilience, and antimicrobial stewardship (AMS) initiatives by creating conditions that can increase healthcare-associated infections (HAIs) and AMR transmission [69]. Damage to infrastructure, supply-chain interruptions, and surge demand after extreme weather events can further reduce WASH (water, sanitation, hygiene) performance and adherence to IPC protocols, amplifying transmission risk [66].

There is a need to adopt IPC and AMR approaches that comprise environmentally friendly practices, and have a high impact on sustainability, and can be implemented without compromising safety, efficiency, or patient outcomes [70]. Policies and operational steps should prioritize affordable, high-impact IPC/WASH and stewardship continuity during climate shocks, environmental surveillance of resistance, and cross-sector collaboration. Therefore, AMS and IPC interventions at the national and healthcare facility levels should be considered as climate change adaptation measures, as they will reduce the burden of AMR, which will be worsened by climate change effects. As a moral imperative, high-emission countries in Europe and America should fund hospital AMS and IPC initiatives, not as aid, but through the lens of climate financing justice.

The United Nations [71] warns that climate change is a “threat multiplier” that degrades the determinants of health and reverses hard-won progress. Sierra Leone’s weak health infrastructure and shortage of healthcare workers mean that even modest increases in climate-related illness could overwhelm the health system. Without urgent adaptation and mitigation measures, climate change effects on disease burden and food security will undermine efforts toward the achievement of Universal Health Coverage and the SDGs by 2030.

## Key adaptation strategies for immediate implementation in Sierra Leone

### Build climate-resilient and adaptive health systems

This must translate into tangible actions linked to measurable indicators and time-bound targets: (1) Human resources: Train 80% of primary healthcare workers in climate-sensitive disease management by 2028; (2) Surveillance: Establish early-warning systems covering 70% of high-risk districts by 2030 that link meteorological forecasts with alerts for cholera, malaria, and other disease outbreaks for proactive public health responses; (3) Infrastructure: Retrofit 50% of health facilities with solar power and flood-resistant infrastructure by 2032. These targets provide a clear implementation matrix for monitoring progress.

### Invest in inclusive and green urban planning

Prioritize the upgrading of informal settlements with improved drainage, clean water access, and waste management, directly involving community members in the planning process. Enforce building codes that prohibit construction in high-risk landslide or flood zones and promote green infrastructure, such as mangrove restoration and urban tree planting. Prioritization of these strategies should be informed by preliminary cost–benefit analyses, with urban upgrading and early-warning systems offering high returns on investment, ensuring resource-efficient allocation within Sierra Leone’s constrained fiscal space. Innovative approaches can further enhance adaptation, including leveraging climate finance through blended mechanisms (e.g. public–private partnerships for green infrastructure), integrating AI-based early warning systems for precise flood predictions, and pursuing legal remedies under the ICJ ruling to secure the Loss and Damage funding for health system resilience, tailored to Sierra Leone’s unique socio-economic context.

### Leveraging the ICJ opinion for action

Sierra Leone’s government and civil society organizations should strategically use the ICJ’s advisory opinion in all climate-related diplomatic and financial negotiations. For example, when seeking funds from the Green Climate Fund or Loss and Damage Fund, proposals for the specific adaptations listed above can be framed not as requests for aid, but as essential actions required to uphold the legally and morally affirmed human right to health, for which high-emitting nations bear historical responsibility. Benchmarking Sierra Leone’s adaptation progress against peers like Ghana, which has advanced community-based flood resilience, and aligning with Global Stock-take outcomes from COP28 (e.g., tripling adaptation finance by 2030), can further guide national efforts and position Sierra Leone within global climate accountability frameworks. The Ministry of Environment and Climate Change, the Ministry of Health, and other national and international health partners should develop a comprehensive 2-year climate adaptation plan that should be presented during the COP 31 in Turkey in 2026 From a standpoint of equity, high-income countries should commit to funding the implementation of the country’s 2-year plan as a legal obligation, not as a voluntary pledge, in relation to the ICJ 2025 opinion. Furthermore, Sierra Leone should demand increased adaptation finance from climate funds (Green Climate Fund, Global Environment Facility) by citing the ICJ opinion’s recognition of its vulnerability and the legal duties of wealthier states. The government of Sierra Leone should strengthen alliances with climate-vulnerable nations, position itself as a regional leader in climate diplomacy, and prioritize adaptation backed by international legal authority.

## Conclusion and recommendations

Climate change is already undermining health, livelihoods, and development in Sierra Leone and other countries in SSA. The interaction between environmental disruption, health inequity, and weak adaptation capacity demands a whole-of-government and whole-of-society response. The 2025 ICJ advisory opinion adds important legal and moral weight to this agenda by reinforcing the duty of States to prevent foreseeable climate harm and protect fundamental human rights, including the right to health.

**Fig. 1:**
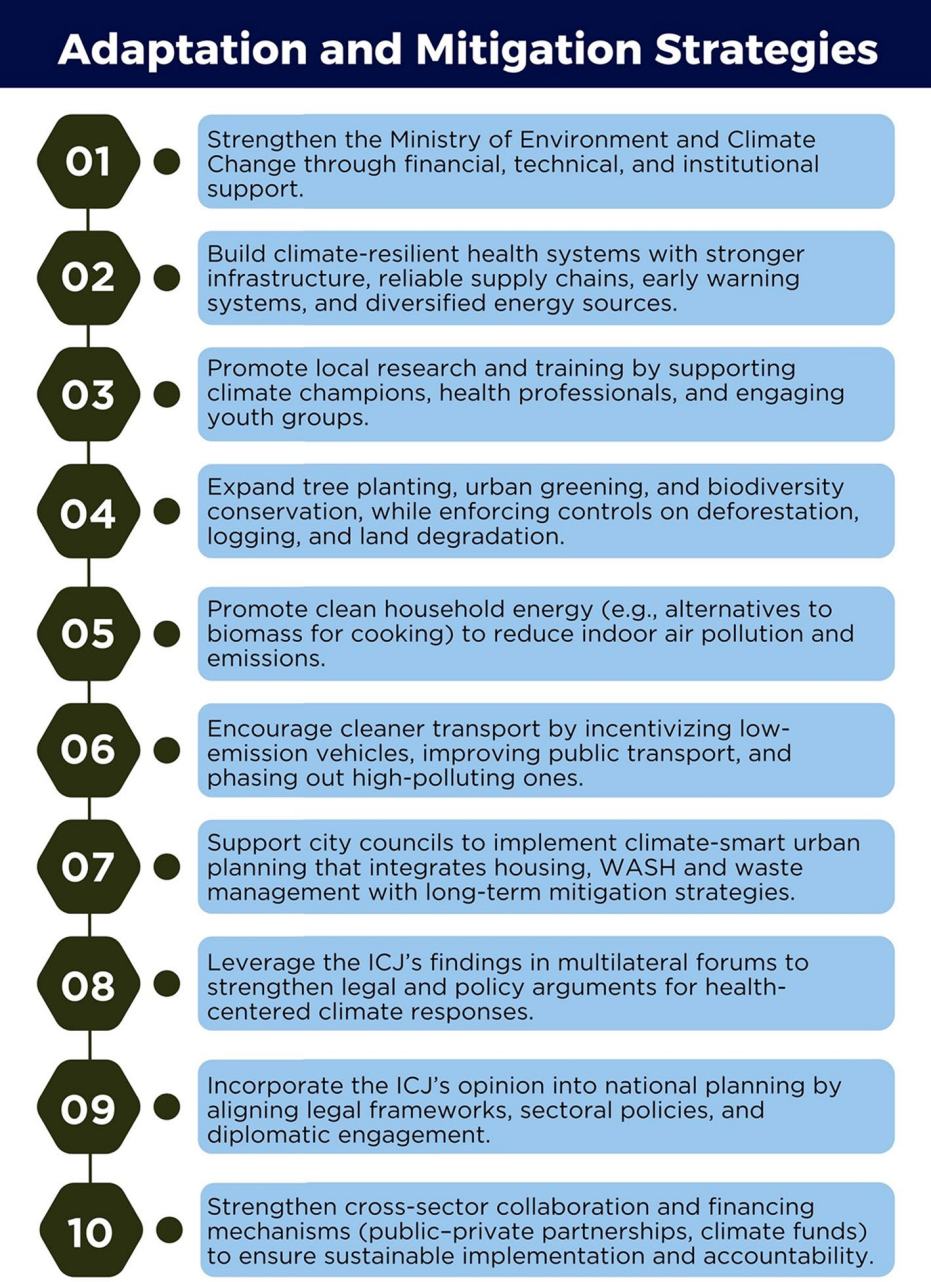
Adaptation and mitigation approaches to climate change in Africa

Based on this analysis, we propose specific, actionable recommendations clustered under adaptation and mitigation strategies that can be implemented in Africa (Fig. 1). These recommendations are designed to be both technically sound and politically feasible within the climate justice framework provided by the ICJ opinion.

## Data Availability

Not applicable.

## Abbreviations

ICJ: International Court of Justice
WHO: World Health Organization
GDP: Gross domestic product
LMICs: Low- and middle-income countries
SSA: Sub-Saharan Africa
IPCC: Intergovernmental Panel on Climate Change
CO_2_: Carbon dioxide
NAPA: National Adaptation Plan for Action
NCDs: Non-communicable diseases
NDC: Nationally Determined Contributions
ANC: Antenatal care
SDGs: Sustainable Development Goals
GHI: Global Hunger Index
UNICEF: United Nations International Children’s Emergency Fund
COP26: 26Th Conference of the Parties (to the UN Framework Convention on Climate Change)
USAID: United States Agency for International Development
UHC: Universal Health Coverage
AMR: Antimicrobial resistance
AMS: Antimicrobial stewardship
IPC: Infection prevention and control
UNFCCC: United Nations Framework Convention on Climate Change

## References

[1] International Court of Justice. Obligations of states in respect of climate change (advisory opinion). ICJ general list no. 187. The Hague: ICJ. https://www.icj-cij.org/case/187/written-proceedings. Accessed 29 Aug 2025.

[2] Robinson SA. Climate change and extreme events are changing the biology of polar regions. Glob Change Biol. 2022;28(20):5861–4. 10.1111/gcb.16309 35821589

[3] Schlosberg D, Collins LB. From environmental to climate justice: climate change and the discourse of environmental justice. Wiley Interdiscip Rev Clim Change. 2014;5(3):359–74.

[4] United Nations. What is climate change? New York: United Nations. https://www.un.org/en/climatechange/what-is-climate-change. Accessed 28 June 2025.

[5] World Health Organization. Climate change. https://www.who.int/news-room/fact-sheets/detail/climate-change-and-health. Accessed 4 Feb 2024.

[6] World Meteorological Organization. Africa suffers disproportionately from climate change. https://wmo.int/media/news/africa-suffers-disproportionately-from-climate-change. Accessed 27 June 2025.

[7] International Trade Administration. Sierra Leone—agriculture sector. https://www.trade.gov/country-commercial-guides/sierra-leone-agriculture-sector. Accessed 28 June 2025.

[8] World Bank Group. Two new World Bank reports offer roadmap for Sierra Leone’s sustainable growth amid climate threats. https://www.worldbank.org/en/news/press-release/2025/06/16/two-new-world-bank-reports-offer-roadmap-for-sierra-leone-s-sustainable-growth-amid-climate-threats. Accessed 6 June 2025.

[9] Sesay U, Osborne A. Building climate-resilient health systems in Sierra Leone: addressing the dual burden of infectious and climate-related diseases. Infect Dis Poverty. 2025;14(1):23. 40122844 10.1186/s40249-025-01294-9 PMC11931751

[10] Siders AR. Adaptive capacity to climate change: a synthesis of concepts, methods, and findings in a fragmented field. Wiley Interdiscip Rev Clim Change. 2019;10(3):e573.

[11] Wolfrum R, editor. Recognition. In: Max Planck Encyclopedia of public international law. Oxford: Oxford University Press. 10.1093/law:epil/9780199231690/law-9780199231690-e1568.

[12] Sattar U. The 4A climate action framework. npj Clim Action. 2024;3(1):103.

[13] World Health Organization. Operational framework for building climate resilient health systems. Geneva: World Health Organization; 2015.

[14] Intergovernmental Panel on Climate Change. Chapter 9: Africa. https://www.ipcc.ch/report/ar6/wg2/chapter/chapter-9/. 28 June 2025

[15] Islam N, Winkel J. Climate change and social inequality. 2017.

[16] Hallegatte S, Rozenberg J. Climate change through a poverty lens. Nat Clim Change. 2017;7(4):250–6.

[17] Sesay U, Osborne A. Sex-disaggregated age-standardized crude suicide rates in Sierra Leone, 2000–2019. Int Health. 2025. 10.1093/inthealth/ihaf137. 41287512 10.1093/inthealth/ihaf137 PMC13530285

[18] Climate and health vulnerability assessment: Sierra Leone. https://www.atachcommunity.com/fileadmin/uploads/atach/Documents/Country_documents/Sierra_Leone_VA_Report_2024.pdf. Accessed 4 Mar 2025.

[19] United Nations. COP26: together for our planet. https://www.un.org/en/climatechange/cop26. Accessed 29 June 2025.

[20] Trisos C, Totin E, Adelekan I, Lennard C, Simpson N, New M, et al. IPCC regional factsheet 3: Central Africa—impacts, adaptation options and investment areas for a climate-resilient Central. https://cdkn.org/sites/default/files/2022-03/IPCC%20Regional%20Factsheet%203_Central%20Africa_web.pdf. Accessed 25 Apr 2024.

[21] Margolis HG. Heat waves and rising temperatures: human health impacts and the determinants of vulnerability. In: Climate change and global public health. Springer; 2020. p. 123–61.

[22] Pörtner HO, Roberts DC, Tignor M, Poloczanska ES, Mintenbeck K, Alegría A, et al., editors. Climate change 2022: impacts, adaptation, and vulnerability. 2022.

[23] Lansana S. Sierra Leone news: at least six dead, hundreds homeless after flash floods hit Freetown. https://premiernews140.medium.com/sierra-leone-news-at-least-six-dead-hundreds-homeless-after-flash-floods-hit-freetown-edf6e0556606. Accessed 29 June 2025.

[24] Government of Sierra Leone. Sierra Leone nationally determined contribution (NDC) 3.o. https://unfccc.int/sites/default/files/2025-12/Sierra%20Leone%20NDC%203.0.pdf. 29 June 2025.

[25] National Academies of Sciences, Engineering, and Medicine, et al. Attribution of extreme weather events in the context of climate change. Washington, DC: National Academies Press; 2016.

[26] Macarthy JM, Koroma B, Rigon A, Frediani AA, Klingel A. Urban transformations in Sierra Leone: knowledge co-production and partnerships for a just city. London: UCL Press; 2024.

[27] Cobbinah PB, Finn BM. Planning and climate change in African cities: informal urbanization and ‘just’ urban transformations. J Plan Lit. 2023;38(3):361–79. 10.1177/08854122221128762.

[28] Johnson MA. An assessment of the urban conditions and systemic issues contributing to slum development in Freetown, Sierra Leone. SLURC working paper. 2009.

[29] World Bank Group. World Bank climate change knowledge portal. https://climateknowledgeportal.worldbank.org/. Accessed 6 July 2025.

[30] Aborode AT, Otorkpa OJ, Abdullateef AO, Oluwaseun OS, Adegoye GA, Aondongu NJ, et al. Impact of climate change-induced flooding water related diseases and malnutrition in Borno State, Nigeria: a public health crisis. Environ Health Insights. 2025. 10.1177/11786302251321683. 40078853 10.1177/11786302251321683 PMC11898090

[31] Laue F, Adegun OB, Ley A. Heat stress adaptation within informal, low-income urban settlements in Africa. Sustainability. 2022;14(13):13. 10.3390/su14138182.

[32] Ramsay EE, Duffy GA, Burge K, Taruc RR, Fleming GM, Faber PA, et al. Spatio-temporal development of the urban heat island in a socioeconomically diverse tropical city. Environ Pollut. 2023. 10.1016/j.envpol.2022.120443. 36265725 10.1016/j.envpol.2022.120443

[33] Muheirwe F, Kihila JM, Kombe WJ, Campitelli A. Solid waste management regulation in the informal settlements: a social-ecological context from Kampala city, Uganda. Front Sustain. 2023;4:1010046.

[34] UN Women. Feminist climate justice: a framework for action. https://www.unwomen.org/sites/default/files/2023-12/Feminist-climate-justice-A-framework-for-action-en.pdf. Accessed 12 Jan 2026.

[35] Atkinson CL. Informal settlements: a new understanding for governance and vulnerability study. Urban Sci. 2024;8(4):158.

[36] Baye F. Exploring urban infrastructure challenges in informal peri-urban Woldia: barriers, implications, and informal strategies. Front Sustain Resour Manag. 2025. 10.3389/fsrma.2025.1555564.

[37] Satterthwaite D, Archer D, Colenbrander S, Dodman D, Hardoy J, Mitlin D, et al. Building resilience to climate change in informal settlements. One Earth. 2020. 10.1016/j.oneear.2020.02.002.

[38] United Nations Human Settlements Programme. The new urban agenda illustrated. Nairobi: UN-Habitat; 2020.

[39] Kidane B, Urugo MM, Hirpha HH, Paulos T, Hundea W, Tessema F. Nutritional challenges of staple crops due to increasing atmospheric carbon dioxide levels: case of Sub-Saharan Africa. J Agric Food Res. 2025;19:101592.

[40] UNICEF. Strengthening nutrition systems to fight against malnutrition in Sierra Leone. https://www.unicef.org/sierraleone/stories/strengthening-nutrition-systems-fight-against-malnutrition-sierra-leone. Accessed 14 July 2025

[41] Fanzo J, McLaren R, Davis C, Choufani J. Climate change and variability: what are the risks for nutrition, diets, and food systems? 2017.

[42] Yila KM, Gboku MLS, Lebbie MS, Kamara LI. Changes in rainfall and temperature and its impact on crop production in Moyamba District, Southern Sierra Leone. Atmos Clim Sci. 2022;13(1):19–43.

[43] van Berkum S, de Steenhuijsen Piters B. Global market and food security in Africa: more trade or more domestic production? In: Pathways to African food security. Routledge; 2025. p. 100–10.

[44] Mutenje MJ, Farnworth CR, Stirling C, Thierfelder C, Mupangwa W, Nyagumbo I. A cost-benefit analysis of climate-smart agriculture options in Southern Africa: balancing gender and technology. Ecol Econ. 2019;163:126–37.

[45] Nkomwa EC, Joshua MK, Ngongondo C, Monjerezi M, Chipungu F. Assessing indigenous knowledge systems and climate change adaptation strategies in agriculture: a case study of Chagaka Village, Chikhwawa, Southern Malawi. Phys Chem Earth. 2014;67:164–72.

[46] Global Hunger Index. Sierra Leone. https://www.globalhungerindex.org/sierra-leone.html. Accessed 13 July 2025.

[47] Morales F, de la Paz SM, Leon MJ, Rivero-Pino F. Effects of malnutrition on the immune system and infection and the role of nutritional strategies regarding improvements in children’s health status: a literature review. Nutrients. 2023. 10.3390/nu16010001. 38201831 10.3390/nu16010001 PMC10780435

[48] Hansen J, Hellin J, Rosenstock T, Fisher E, Cairns J, Stirling C, et al. Climate risk management and rural poverty reduction. Agric Syst. 2019. 10.1016/j.agsy.2018.01.019.

[49] World Health Organization. Everybody’s business: strengthening health systems to improve health outcomes: WHO’s framework for action. https://www.who.int/healthsystems/strategy/everybodys_business.pdf. Accessed 12 Jan 2026.

[50] Elston J, Moosa A, Moses F, Walker G, Dotta N, Waldman RJ, et al. Impact of the Ebola outbreak on health systems and population health in Sierra Leone. J Public Health. 2016;38(4):673–8. 10.1093/pubmed/fdv158 28158472

[51] Watts N, Amann M, Ayeb-Karlsson S, Belesova K, Bouley T, Boykoff M, et al. The Lancet Countdown on health and climate change: from 25 years of inaction to a global transformation for public health. Lancet. 2018;391(10120):581–630. 29096948 10.1016/S0140-6736(17)32464-9

[52] Haines A, Ebi K. The imperative for climate action to protect health. N Engl J Med. 2019;380(3):263–73. 30650330 10.1056/NEJMra1807873

[53] Martins FP, Paschoalotto MAC, Closs J, Bukowski M, Veras MM. The double burden: climate change challenges for health systems. Environ Health Insights. 2024. 10.1177/11786302241298789. 39575137 10.1177/11786302241298789 PMC11580064

[54] Musafiri S, Masaisa F, Bavuma M, Kalisa U, Rutayisire P. Indoor air pollution from cooking with biomass fuels is a major cause of chronic bronchitis among women in a rural district of Rwanda. Afr J Respir Med. 2018;14(1):1–4.

[55] Natur S, Damri O, Agam G. The effect of global warming on complex disorders (mental disorders, primary hypertension, and type 2 diabetes). Int J Environ Res Public Health. 2022;19(15):9398. 35954764 10.3390/ijerph19159398 PMC9368177

[56] Megersa DM, Luo XS. Effects of climate change on malaria risk to human health: a review. Atmosphere. 2025. 10.3390/atmos16010071.

[57] World Health Organization. Experts warn of serious health impacts from climate change for pregnant women, children, and older people. https://www.who.int/news/item/05-06-2024-experts-warn-of-serious-health-impacts-from-climate-change-for-pregnant-women--children--and-older-people. Accessed 14 July 2025. PMC11237281 38955455

[58] Proulx K, Daelmans B, Baltag V, Banati P. Climate change impacts on child and adolescent health and well-being: a narrative review. J Glob Health. 2024;14:04061. 38781568 10.7189/jogh.14.04061 PMC11115477

[59] Baird S, Choonara S, Azzopardi PS, Banati P, Bessant J, Biermann O, et al. A call to action: the second Lancet Commission on adolescent health and wellbeing. Lancet. 2025;405(10493):1945–2022. 40409329 10.1016/S0140-6736(25)00503-3

[60] Sbiroli E, Geynisman-Tan J, Sood N, Maines BA, Junn JHJ, Sorensen C. Climate change and women’s health in the United States: impacts and opportunities. J Clim Change Health. 2022;8:100169. 10.1016/j.joclim.2024.100331 PMC12851335 41647128

[61] Clayton S. Climate anxiety: psychological responses to climate change. J Anxiety Disord. 2020;74:102263. 32623280 10.1016/j.janxdis.2020.102263

[62] Chersich MF, Maimela G, Lakhoo DP, Solarin I, Parker C, Scorgie F. Climate change impacts on maternal and new-born health in Africa: intervention options. Wits J Clin Med. 2022. 10.18772/26180197.2022.v4n3a7.

[63] Dumbuya S, Chabinga R, Ferede MA, Saber M. Climate change impacts on maternal health and pregnancy outcomes in Africa. J Water Health. 2024. 10.2166/wh.2024.254. 39611672 10.2166/wh.2024.254

[64] van Bavel B, Berrang-Ford L, Moon K, Gudda F, Thornton AJ, Robinson RFS, et al. Intersections between climate change and antimicrobial resistance: a systematic scoping review. Lancet Planet Health. 2024;8(12):e1118–28. 10.1016/S2542-5196(24)00273-0. 39674199 10.1016/S2542-5196(24)00273-0

[65] Fernández Salgueiro C, Cernuda Martínez, Gan RK, Arcos González. Climate change and antibiotic resistance: a scoping review. 10.1111/1758-2229.70008. 10.1111/1758-2229.70008 PMC11393301 39267332

[66] Abeles SR, Kline A, Lee P. Climate change and resilience for antimicrobial stewardship and infection prevention. Curr Opin Infect Dis. 2024;37(4):270–6. 38843434 10.1097/QCO.0000000000001032

[67] Freifeld AG, Todd AI, Khan AS. The climate crisis and healthcare: what do infection prevention and stewardship professionals need to know? Antimicrob Steward Healthc Epidemiol. 2023;3(1):e136. 37592967 10.1017/ash.2023.170 PMC10428152

[68] Lim O, Chua WY, Wong A, Ling RR, Chan HC, Quek SC, et al. The environmental impact and sustainability of infection control practices: a systematic scoping review. Antimicrob Resist Infect Control. 2024;13(1):156. 39719628 10.1186/s13756-024-01507-0 PMC11668097

[69] Graham SB, Machalaba C, Baum SE, Raufman J, Hill SE. Applying a One Health lens to understanding the impact of climate and environmental change on healthcare-associated infections. Antimicrob Steward Healthc Epidemiol. 2023;3(1):e93. 37228504 10.1017/ash.2023.159 PMC10204136

[70] Lee PS, Frantzis I, Abeles SR. Greening infection prevention and control: multifaceted approaches to a sustainable future. Open Forum Infect Dis. 2024;12(2):ofae371. 39958523 10.1093/ofid/ofae371 PMC11825990

[71] United Nations Security Council. La Commission de consolidation de la paix. 2024 (**in French**).

